# Neonatal intestinal dysbiosis in necrotizing enterocolitis

**DOI:** 10.1186/s10020-018-0002-0

**Published:** 2018-03-15

**Authors:** Naomi-Liza Denning, Jose M. Prince

**Affiliations:** 1 Division of Pediatric Surgery, Zucker School of Medicine at Hofstra/Northwell, Cohen Children’s Medical Center, 269-01 76th Avenue, CH 158, New Hyde Park, New York, NY 11040 USA; 2 Feinstein Institute for Medical Research, Manhasset, NY 11030 USA; 3 Trauma Institute, Northwell Health System, Manhasset, NY 11030 USA

**Keywords:** Neonatal sepsis, Microbiome, Intestinal failure, Prematurity, Inflammation

## Abstract

Necrotizing Enterocolitis (NEC) is one of the most devastating gastrointestinal diseases in neonates, particularly among preterm infants in whom surgical NEC is the leading cause of morbidity. NEC pathophysiology occurs in the hyper-reactive milieu of the premature gut after bacterial colonization. The resultant activation of the TLR4 pathway appears to be a strongly contributing factor. Advancements in metagenomics may yield new clarity to the relationship between the neonatal intestinal microbiome and the development of NEC. After a century without effective directed treatments, microbiome manipulation offers a promising therapeutic target for the prevention and treatment of this devastating disease.

## Background

**Fig. 1:**
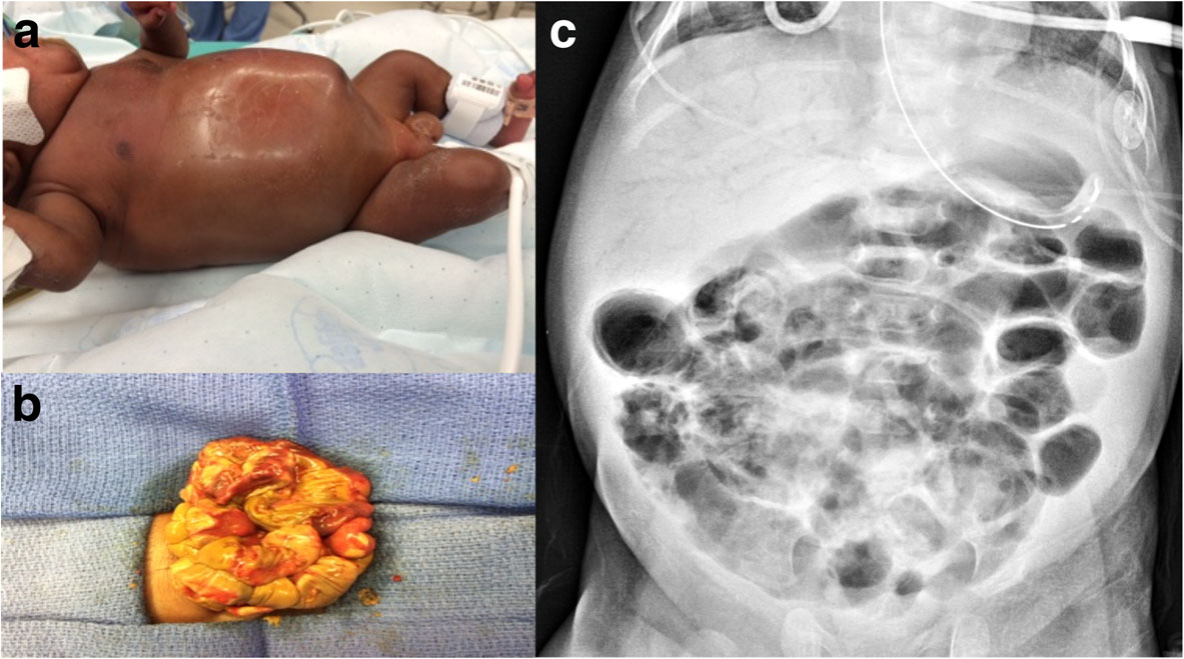
Clinical findings of Necrotizing Enterocolitis. **a** Abdominal distension and erythema frequently seen in an infant with necrotizing enterocolitis. **b** Necrotic bowel found upon surgical exploration for necrotizing enterocolitis. **c** Abdominal radiograph demonstrating portal venous gas and pneumatosis intestinalis

Worldwide, sepsis is the third leading cause of neonatal mortality (Hansen et al. 2017a). Necrotizing Enterocolitis (NEC) is one of the most devastating gastrointestinal diseases in neonates, particularly among preterm infants in whom surgical NEC is the leading cause of morbidity (Carter and Holditch-Davis 2008). NEC is characterized by submucosal edema and hemorrhage, infiltration of the intestinal wall by neutrophils, disruption of the intestinal villus architecture, and in severe cases, full thickness necrosis or intestinal wall perforation (Papillon et al. 2013). Classic early clinical signs of the disease include abdominal distension, feeding intolerance, and bloody stool in infants around 1 week old. Abdominal radiographs can demonstrate pneumatosis intestinalis and/or portal venous gas (Neu and Walker 2011) (Fig. 1).

In North America, NEC occurs in about 7% of infants born between 500 and 1500 g (Neu and Walker 2011) which translates into an incidence of around 1.1 per 1000 live births (Papillon et al. 2013). NEC has a mortality rate of approximately 30 %; lower birth weight infants and infants who require surgical treatment of NEC experience a higher mortality rate than larger babies or infants in whom NEC can be managed medically (McElroy 2014; Fitzgibbons et al. 2009; Abdullah et al. 2010). Necrotizing enterocolitis costs the United States health care system over one billion dollars per year (McElroy 2014) with an average cost for surgical NEC between 300,000 and 600,000 dollars per patient (Stey et al. 2015). In addition to the immediate morbidity and economic costs associated with NEC, the disease results in long term sequel in around 25% of the time, such as neurodevelopmental delays or short gut syndrome (Neu and Walker 2011; Nino et al. 2016; Wadhawan et al. 2014).

## The neonatal immune system and necrotizing enterocolitis

NEC is a disease that occurs predominately in premature infants; the likelihood of developing NEC is inversely proportional to birth weight and gestational age (Guthrie et al. 2003). Interestingly, the onset of NEC appears to be most related to post gestational age (corrected postnatal age) as opposed to actual postnatal age. The peak incidence of NEC seems to occur at approximately 31 weeks post conceptual age. This highlights the relationship between host development and the development of necrotizing enterocolitis (Neu and Pammi 2017). There are several key differences between the preterm and the term neonate that contribute to the increased propensity of preterm neonates to develop NEC. The gastrointestinal tract of the preterm neonate demonstrates decreased intestinal barrier function (Xing et al. 2017; Moore et al. 2016), an impaired intestinal immune defense system (Lu et al. 2014b), and an increased inflammatory propensity (Ferretti et al. 2017; Nanthakumar et al. 2011). Furthermore, the immune system of a preterm neonate is less developed than a baby born at term. In all neonates both the adaptive and the innate components of the immune system are immature owing to reduced physical barriers and impaired and delayed function of most cell types (Camacho-Gonzalez et al. 2013; Wynn et al. 2009). Compared to term neonates, preterm infants have a stunted immune system possessing a smaller quantity of monocytes and neutrophils. The quality of these cells is also impaired with a reduced ability to kill pathogens. In addition, preterm neonates’ ability to produce cytokines is lowered translating into limited T cell activation (Table 1) (Melville and Moss 2013; Mussi-Pinhata and Rego 2005).

**Table 1:** Comparison of the Term and Premature Neonatal Immune System

Term	Preterm
↓ Physical barriers	↓↓↓ Physical barriers
↑ Effectiveness of immune cells to target pathogens	↓ Number of monocytes and neutrophils
	↓ Overall ability to produce cytokines
	↓ T cell activation
	↓ Number of natural killer cells
↓ Bactericidal/permability-increasing protein	↓↓ Bactericidal/permability-increasing protein
	↓ Passive Immunity (level of IgG depends on transplacental transfer and thus increases with gestation age)

↑ indicates increased; ↓ indicates decreased

## The development of necrotizing enterocolitis

Despite decades of investigation into the pathophysiology of NEC, it still not well defined. The importance of bacterial colonization in the development of NEC was recognized decades ago by Santulli et al. (Santulli et al. 1975) Despite this no single causative agent has been identified. As such, most theories on the pathogenesis of NEC focus on not a specific pathogen but a generalized microbial imbalance of intestinal flora called dysbiosis (Elgin et al. 2016). One evolving school of thought is that the disruption of normal neonatal intestinal bacterium, or microbiome, induces a proinflammatory state, allowing bacterial translocation across intestinal epithelia (Patel and Denning 2015). In 2016 Nino et al. eloquently proposed a “unifying hypothesis for the development of NEC: that the intestine of the premature neonate exists in a hyper-reactive state relative to the full-term intestine, which favors NEC development upon colonization with an appropriate microbial milieu in a patient with a permissive genetic background” (Nino et al. 2016).

## The neonatal microbiome

The microbiota of each individual is approximately 10–100 trillion microbial cells with the majority of colonization occurring in the gastrointestinal tract (Ursell et al. 2012). The ability to study these cells has greatly expanded in the recent past. Previous methods of evaluation were limited to culture-based techniques with the unfortunate limitation that many bacterial cells seen in feces cannot currently be cultured in a laboratory. In recent years new initiatives such as the Human Microbiome Roadmap, made possible by the development of high throughput molecular techniques to analyze microbial DNA and RNA, have greatly expanded our knowledge of the microbiome (Torrazza and Neu 2013). In a healthy individual the intestinal microbiota is commensal and plays an important role in the regulation of multiple metabolic, immune, and inflammatory pathways within the host (Torrazza and Neu 2013; Hooper and Gordon 2001; Murgas Torrazza and Neu 2011; Round and Mazmanian 2009). This commensal relationship still needs to develop in the neonate, especially the preterm neonate. Furthermore, the premature infant’s underdeveloped immune system has limited ability to defend against harmful bacteria and tolerate commensal species of bacteria (Murgas Torrazza and Neu 2011).

## The development of the neonatal microbiome

Normal term infants are first colonized by *Streptococcus*, *Staphyococcus*, *Escherichia coli*, *Lactobacillus*, and *Enterobacter*. As these species consume oxygen they allow for the subsequent colonization of anaerobic bacterial species, mainly *Clostridia*, *Bifidobacterium*, and members of the *Firmicutes* phyla (Palmer et al. 2007; Park et al. 2005). The intestinal microbiome in premature infants develops differently. Preterm infants are fed enterally earlier than nature intended and frequently receive fortified feeds. They have an immature immune system and frequently have indwelling supportive devices (Elgin et al. 2016). When the gut microbiome of preterm infants was compared to the microbiome of term infants it demonstrated higher levels of facultative anaerobes and reduced levels of anaerobes such as *Bifidobacterium* and *Bacteroides* (Arboleya et al. 2017). In addition they have an increased amount of potentially pathogenic bacteria such as *Escherichia coli*, *Staphylococcus*, and *Klebsiella* (Table 2) (Schwiertz et al. 2003).

**Table 2:** Comparison of the Microbiome of Term and Premature Neonates

Term	Premature
*Clostridia*	↑facultative anaerobes
*Bifidobacterium*	↓*Bifidobacterium*
Other *Firmicutes*	↑*Staphylococcus*
	↑*Escherichia coli*, *Klebsiella* and other *Enterobacteriacease*
	↓*Bacteroidetes*

↑ indicates increased; ↓ indicates decreased

The development of the microbiome in the neonatal gut occurs in two waves. The first wave is common to both term and preterm infants and depends on the mode of childbirth. The factors affecting the second wave vary between term and preterm infants. In term infants the second wave is influenced by type of feeding – breast feeding or formula feeding. As described above, a breast fed infant’s microbiome is rich in *Bifidobacteria* and *Bacteriodes*. In contrast, formula-fed infants remain predominately colonized by *Streptococci*, *Staphylococci* and *Lactobacilli*. Preterm infants’ microbiome is largely characterized by high numbers of *Clostridiaceae* and *Enterobacteriaceae* with low number of *Bifidobacteria* and *Bacteroidetes* (Vongbhavit and Underwood 2016; Arboleya et al. 2012). Overall, the single most important factor in the establishment of the microbiome of preterm infants appears to be the degree of prematurity (La Rosa et al. 2014). Rosa et al. characterized the progression of bacterial colonization in neonates and found that the microbiome developed in an orderly fashion and that mode of delivery, antibiotics, diet, and gestational age of the infant all altered the pace of the progression but not the sequence. They postulate that gut bacterial communities have a nonrandom assembly that is punctuated by microbial population abruptions. They collected all stools (922 specimens) from 58 premature infants, none of which had serious intra-abdominal pathology, weighing < 1500 g in a single tertiary care center neonatal intensive care unit where extensive protocols were in place to limit exposure to microbes to the extent possible. They found that *Bacilli*, *Gammaproteobacteria*, and *Clostridia* represented 91.7% of all bacterial sequences. Only two infants produced stools where those three classes did not predominate (La Rosa et al. 2014).

## The impact of the microbiome on inflammation and the role of toll-like receptors

The intestinal mucosa recognizes bacterial products via pattern recognition receptor (PRRs), the most well studied of which are Toll-Like Receptors (TLR). The TLRs recognize microbial associated molecular patterns (MAMPs) (Rhee 2011). Patterns of intestinal colonization help to regulate TLR expression. As a result, abnormal colonization patterns can trigger inappropriate responses. MAMPs activate specific TLRs which lead to activation of nuclear factor kappa-beta (NF-κβ) and its inflammatory pathway and caspases. These in turn propagate apoptosis and activate transcription genes and induce cytokines (IL-1, IL-6, IL-8, TNF-α, INF-1) (Torrazza and Neu 2013). Although many commensal bacterial do not express some key virulence factors (Murgas Torrazza and Neu 2011) both commensal and pathogenic intestinal microbes contain MAMPS. Even normal flora can be pro-inflammatory if host conditions are abnormal. TLRs must maintain a delicate balance between an appropriate inflammatory response to pathogenic bacteria and homeostasis by supporting important intestinal functions including cell growth and proliferation, cytoprotection, antimicrobial peptide secretion, and regulation of barrier function (Patel and Denning 2015).

After birth, the neonatal gut receives its first large scale challenge of MAMPs activating TLR (Lu et al. 2014a). In addition, the lipopolysaccharides from gram-negative bacteria activate TLR-4 (Neal et al. 2006). After TLR-4 activation, the premature gut, compared to the gut of a full-term neonate, is more likely to mount a relatively increased cytokine-mediated inflammatory response. Among the most prominent of cytokines is interleukin-8 which increases neutrophil chemotaxis and results in inflammation which can lead to tissue injury and NEC (Markel et al. 2006; Sharma et al. 2007). Some investigators believe that the premature neonate’s propensity for an exaggerated inflammatory response is due to a deficiency in the expression of inhibitors of the NF-κβ pathway (Claud et al. 2004; Spehlmann and Eckmann 2009). Unlike pathogenic bacteria, commensal bacteria reside in the gut without triggering inflammation. Commensal microbes help the gut tolerate the constant stimulation occurring in the gastrointestinal tract by preventing their recognition by TLRs or downregulating the NF-κβ pathway by blocking degradation of its inhibitors. In addition, in the normal neonatal gut, the low level stimulus of TLR4 by commensal bacteria is beneficial in intestinal homeostasis (Neish 2004; Neish et al. 2000).

## Toll-like-receptors, inflammation, and necrotizing enterocolitis in the premature gut

Toll like receptor 4 (TLR4) plays a critical role in the development of NEC – its activation leads to mucosal injury and reduced epithelial repair. Furthermore, TLR4 is upregulated in the premature gut as compared to the gut of the full term neonate (Sodhi et al. 2010; Hackam et al. 2013). TLR4 has an important role in the regulation of normal gut development in utero; levels of TLR4 expression typically fall throughout gestation (Gribar et al. 2009). As a result, TLR4 levels are high in preterm neonate. When the gut is subsequently colonized with numerous gram negative bacteria, there are deleterious consequences of exaggerated TLR4 signaling including increased release of proinflammatory cytokines, increased enterocyte apoptosis, and impaired mucosal healing. In addition, bacterial translocation through the gut mucosa activate TLR4 on the endothelia of the intestinal vasculature, resulting in reduction of blood flow and development of intestinal ischemia and necrosis (Nino et al. 2016). A 2017 study by Hui et al. demonstrated increased pro-inflammatory cytokines and enhanced expression of TLR4 in resected intestinal samples from 28 to 29 week old infants with NEC (Hui et al. 2017).

**Fig. 2:**
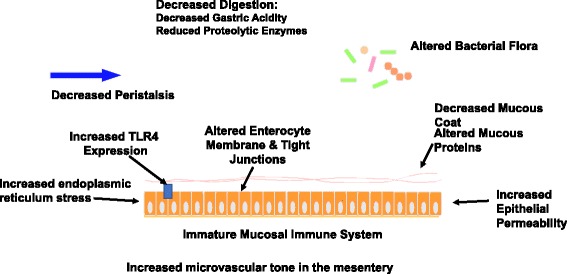
Factors that Predispose the Immature Gut to Necrotizing Enterocolitis. Pictorial representation of factors contributing to the propensity of the immature gut to develop necrotizing enterocolitis

In addition to increased TLR4 signaling there are other factors that predispose the premature gut to the development of NEC (Fig. 2). The premature gut displays decreased digestion, decreased nutrient absorption (Reisinger et al. 2014) and impaired intestinal motility (Ren et al. 2017). It also has a high baseline level of cellular endoplasmic reticulum stress. This increases the likelihood of apoptosis in the intestinal epithelium. Furthermore, there are decreased physical barriers in the premature gut, with a decreased number of mucus-producing goblet cells (Deplancke and Gaskins 2001), immature tight junctions (Anand et al. 2007), and increased microvascular tone in the intestinal mesentery (Watkins and Besner 2013). Although outside of the scope of this review, in addition to the TLR4 pathway, other pathways and cell types are thought to be important in the development of NEC including platelet-activating factor and macrophages (Caplan et al. 2005; Frost and Caplan 2013; Furukawa et al. 1993; Rabinowitz et al. 2001; Maheshwari et al. 2011; MohanKumar et al. 2016).

## The microbiome in necrotizing enterocolitis

Several studies validate the notion that the microbiome of the neonate with NEC is fundamentally different from the microbiome of the neonate who is unaffected by NEC. However, there are a range of organisms implicated in these studies, further highlighting the lack of a single causative agent. Additionally, direct comparison of these studies are difficult due to limitations in 16S rRNA sequencing (speciation is dependent on the quality and length of the sequence, challenging primer design, and inability to distinguish between living and dead bacteria) and heterogeneous populations studied- including a wide range of post gestational ages at which NEC develops (Hosny 2017).

Despite these limitations studies investigating the microbiome of a neonate with NEC have been informative. Among those studies, Wang et al. reported a study of 20 preterm infants from a single institution, 10 suffering from NEC and 10 without the disease. These patients included four twin pairs. Bacterial DNA from fecal samples were obtained and underwent sequencing of the 16S rRNA gene. All 20 infants had low levels of diversity in the intestinal bacterial colonization but patients with NEC had a significantly reduced level of diversity compared to unaffected neonates. They had an increase in the colonization of *Gammaproteobacteria* with a decrease in other bacterial species (Wang et al. 2009). Mai et al. collected weekly stool samples from infants with a gestation age < 32 weeks or a birth weight ≤ 1250 g. They then used 16S rRNA sequencing to compare the diversity of the microbiota and the prevalence of specific bacteria in nine infants with NEC and nine matched controls. Patients with NEC has an increase in *Proteobacteria* and a decrease in *Firmicutes* between 1 week and < 72 h prior to the detection of clinical NEC (Mai et al. 2011).

Investigators have also searched for a microbial pattern that appears prior to NEC onset. Morrow et al. analyzed stool samples from infants < 29 weeks gestational age and compared infants who developed NEC to matched controls. Infants who developed NEC not only had lower diversity in their microbiome but distinct patterns. In postnatal days 4 to 9, infants who developed NEC were dominated by members of the *Firmicutes* phylum. During days 10 to 16, samples from the remaining NEC cases were dominated by *Proteobacteria*. Interestingly, infants with *Firmicutes* dysbiosis developed NEC earlier than infants with *Proteobacteria* dysbiosis. All infants with NEC lacked *Propionibacterium* and were preceded by either *Firmicutes* or *Proteobacteria* dysbiosis. However, it should be noted that 25% of controls had this phenotype as well (Morrow et al. 2013). Multiple studies have shown that *Proteobacteria* can be associated with an increased incidence of NEC; a fact that has been validated in the 2017 meta-analysis of 14 previous studies of intestinal dysbiosis in preterm infants who subsequently developed NEC by Pammi and et al. (Lu and Ni 2015; Gritz and Bhandari 2015; Pammi et al. 2017).

## Factors impacting the neonatal microbiome

**Fig. 3:**
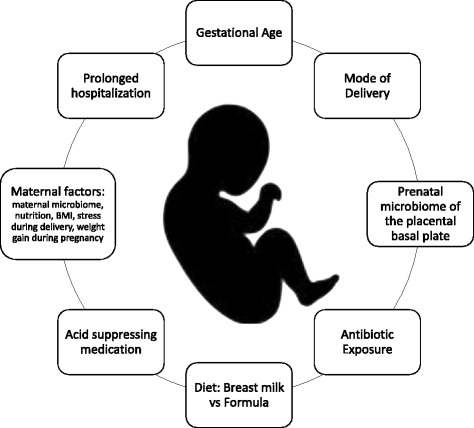
Factors Impacting the Neonatal Gut Microbiome. Factors contributing to the development of the neonatal microbiome include both prenatal factors such as the maternal microbiome, the microbiome of the amniotic fluid, the degree of prematurity and the mode of delivery, and postnatal exposures including antibiotics, diet, and acid suppressing medications

Given the differences in the microbiota of infants with NEC, we will now consider factors that predispose premature infants to dysbiosis focusing on the degree of prematurity, antibiotics, formula feeding, and acid suppressing medications (Fig. 3) (Vongbhavit and Underwood 2016). Other factors predisposing to dysbiosis, but not yet convincingly demonstrated to be associated with the development of NEC include mode of delivery and environmental toxins (Madan et al. 2012; Moya-Perez et al. 2017). Additionally, the impact of the NICU environment on the premature infants’ microbiome has not been fully established. Studies have established the diversity in the surface microbiota of various NICUs and demonstrated that intensive cleaning is effective in reshaping but not eliminating the microbiota (Underwood and Sohn 2017).

## Prenatal development of the microbiome

PCR studies of amniotic fluid have estimated the prevalence of microbial invasion of the amniotic cavity to be more than 30–50% higher than previously detected by culture based methods (DiGiulio 2012). The placental basal plate was found to have a microbiome of its own with many commensal bacterial species including organisms from the phyla *Firmicutes*, *Tenericutes*, *Proteobacteria*, *Bacteriodetes*, and *Fusobacteria* (Aagaard et al. 2014). It is unclear whether this colonization has any impact on the neonatal GI tract but, given that the fetus swallows large volumes of amniotic fluid during gestation, it is logical that the fetal intestine would be exposed to amniotic fluid microbes (Torrazza and Neu 2013). This notion is further supported by the findings of low levels of microbial DNA in first-pass meconium (Hansen et al. 2015; Nagpal et al. 2016). Jimenez et al. were able to isolate low numbers of *Enterococcus*, *Staphylococcus*, and *Streptococcus* in the umbilical blood from scheduled, elective cesarean sections. In a later study they tested the meconium from term infants prior to breast feeding and found similar organisms: *Enterococcus*, *Staphylococcus*, and *Escherichia coli* (Jimenez et al. 2008; Jimenez et al. 2005).

## The impact of mode of delivery on the microbiome

In the United States the caesarean section rate continues to rise, reaching 33.1% in 2013 (Mistry et al. 2006). Several studies have demonstrated a difference in the microbiome of infants born via cesarean delivery compared to vaginally delivered neonates. Infants born via the vaginal canal are typically seeded with vaginal flora including *Lactobacillus* and *Prevotella*. In contrast, infants born via cesarean section are typically seeded with skin flora (Biasucci et al. 2008). Infants born via cesarean section display delayed onset of colonization of *Bifidobacterium* and *Bacteroides* with increased levels of colonization by the *Enterobacteriaceae* family (Dogra et al. 2015). In 2011 Domingiuez-Bello et al. used sequencing technology to demonstrate that the gastrointestinal microbiota of infants born vaginally were colonized with *Lactobacillus*, but infants born via cesarean delivery were colonized by bacteria typically found in skin and hospitals such as *Staphylococcus* and *Acinetobacter* (Dominguez-Bello et al. 2011). They later demonstrated that exposing neonates delivered via cesarean section to maternal vaginal fluids at birth could redirect the microbiome, making it similar to neonates delivered vaginally (Dominguez-Bello et al. 2016). Large numbers of epidemiologic studies have demonstrated compelling evidence suggesting a link between cesarean delivery and increased risk of obesity, asthma, allergies, immune deficiencies, and other atopic disease (Thavagnanam et al. 2008; Pistiner et al. 2008; Huh et al. 2012; Sevelsted et al. 2015). However, to date, a direct link between delivery by cesarean and NEC has not been found. Prognostic studies indicate that cesarean section is a risk factor for NEC but this is likely correlated not causative (Samuels et al. 2017).

## Dietary impact on the microbiome

Multiple studies over several decades have demonstrated that enteral feeding with human milk as opposed to formula decreases the incidence of NEC (Sullivan et al. 2010; Lucas and Cole 1990). Breast milk contains immunoglobulins, cytokines, lactoferrin, and growth factors (Torrazza and Neu 2013). Breast milk also contains glycoproteins that have been shown to decrease organ injury and inflammation in sepsis in mouse models (Hansen et al. 2017b). In addition human milk contains human milk oligosaccharides (HMO) that stimulates the growth of “healthy” bacteria- *Bifidobacteria* and *Bacteroides* species both possess the proper enzymes to digest HMOs and metabolize them for energy. HMOs are the third most abundant ingredient in breast milk (Elgin et al. 2016). HMOs may help to select for beneficial microbes by providing them with substrates for growth, allowing them to thrive. This may decrease the ability of opportunistic pathogenic microbes to gain a foothold in the neonatal gut (Elgin et al. 2016). Furthermore, one way in which breast milk is thought to be beneficial is downregulation of TLR4 signaling (He et al. 2016; Good et al. 2015).

In addition to helping shape the intestinal microbiome by nutrient selection, breast milk has its own microbiome which evolves over time. Initially colostrum contains *Staphylococcus*, *Streptococcus*, *Lactobacillus* and *Weissella* but over time the microbes are more consistent with maternal oral flora (*Veillonella*, *Leptotrichia*, and *Prevotella*). Interestingly, while milk samples from mothers who underwent elective cesarean sections varied in bacterial composition from milk samples from mothers who experienced vaginal delivery, the microbiome in the breast milk of mothers who underwent nonelective cesarean sections was similar to the microbiome of milk among mothers with vaginal deliveries. This suggests that maternal stress and hormones influence breast milk microbiome more directly than mode of delivery (Cabrera-Rubio et al. 2012).

After birth, breast fed infants are first colonized with aerobic or facultative anaerobic bacteria followed by a bloom of anaerobic bacteria. Formula fed infants’ gastrointestinal microbiomes differ by having fewer anaerobes and a plethora of gram negative bacteria (Le Huerou-Luron et al. 2010) and have increased levels of *Enterobacteriaceae*, *Bacteroides*, and *Clostridium* in their stools compared to infants who receive breast milk. The effect on breast versus formula feeding on the levels of the *Bifidobacterium* species are less clear with some studies finding significantly reduced amounts in formula fed infants and other studies showing no difference at all (Penders et al. 2005; Roger et al. 2010; Tannock et al. 2013; Adlerberth and Wold 2009; Bezirtzoglou et al. 2011).

## Impact of antibiotics on the microbiome

Antibiotic exposure has a large impact on the neonatal microbiome delaying the colonization of beneficial bacteria and reducing the diversity of the intestinal microbiome, both factors which are thought to predispose the neonate to NEC (Torrazza and Neu 2013). Years of research and numerous studies have demonstrated that use of antibiotics may be associated with development of NEC (Cotten et al. 2009; Abdel Ghany and Ali 2012; Kuppala et al. 2011). Alexander et al. demonstrated there was a direct correlation between duration of antibiotics and risk of developing NEC among infants without culture-proven sepsis (Alexander et al. 2011). For more detailed review of the topic, Esaiassen et al. published a meta-analysis in 2017 demonstrating the same: prolonged antibiotic exposure in uninfected preterm infants is associated with an increased risk of NEC and/or death (Esaiassen et al. 2017).

## Impact of acid suppression on the microbiome

Acid suppression therapy has a known impact on the preterm microbiome. Gupta et al. demonstrated that the use of H2 blockers in premature infants shifts the microflora pattern towards *Proteobacteria* and limits the diversity of the fecal microbiome. These alterations may predispose an infant to NEC (Gupta et al. 2013). Romaine et al. performed a retrospective cohort study and found that the use of H2 blockers are associated with increased risk of the combined outcome of death, NEC, or sepsis in hospitalized very low birth weight infants (Romaine et al. 2016).

## Therapeutic alteration of the neonatal microbiome

Given the link between dysbiosis and NEC, altering the microbiome is a promising target for future therapies (Vongbhavit and Underwood 2016). A 2014 Cochrane review of randomized and quasi-randomized trials found that enteral supplementation of probiotics prevents severe NEC and all cause mortality in preterm infants (Robinson 2014). In 2016 Denkel et al. found that dual-strain probiotics reduced NEC and mortality in preterm infants in a German NICU (Denkel et al. 2016). However, the evidence regarding probiotics is difficult to interpret. Although the meta-analyses of probiotics usage have shown a beneficial effect, not all individual randomized control trials have demonstrated the same. Trials are difficult to generalize as many use a different study design, differing probiotics, and differing infant diets and feeding times (Patel et al. 2017). The strain of probiotics used is likely to be important. The PiPs trial did not demonstrate any benefit with routine administration of *Bifidobacterium breve* (Costeloe et al. 2016). Furthermore, there are conflicting opinions regarding giving live bacteria to particular vulnerable preterm neonates.

## Future directions

Research over the last decade has demonstrated the importance of the gut microbiome on human health and disease. Microbiome alterations have been associated with a vast array of diseases ranging from cardiovascular disease to colorectal cancer, obesity, diabetes, and rheumatoid arthritis (Shreiner et al. 2015). Furthermore, microbiome manipulation has already proven beneficial in the treatment of clostridium difficile infection (Brandt 2012) and has demonstrated promising results in the treatment of inflammatory bowel disease (Hansen and Sartor 2015) and in experimental models of obesity (Jayasinghe et al. 2016). The above review demonstrates the link between gut dysbiosis and necrotizing enterocolitis. It is logical then, that future prevention and treatment of the disease will also include a component of microbiome manipulation.

There are three major options for an approach to microbiome-based therapies: additive, subtractive, or modulatory therapies. Additive therapy includes the manipulation of the microbiome by supplementing the microbiome of the host with either specific strains of organisms or groups of natural or engineered microorganisms. Subtractive therapy involves the removal of specific deleterious members of the microbiome to cure disease. Modulatory therapies involve administration of nonliving agents, called prebiotics, to modify the composition or activity of the host microbiome (Mimee et al. 2016).

Probiotics are discussed in detail above. However, before probiotics can routinely be used in the prevention of NEC, dose, strain, and timing of administration need to be standardized. Probiotics might require regulatory approval for use in the neonate before they can become standard of care. In addition to commercially available probiotics the development of genetically engineered probiotics are underway, although this process is still in its infancy. Bacterial cells could be altered to allow recombinant expression of therapeutic biomolecules. This would overcome issues with bioavailability and drug inactivation with oral administration. Protein synthesis of the therapeutics could be tied to conditions associated with the disease (Mimee et al. 2016).

Quantitative metagenomics can be used to directly map the human gut microbiome. In the future this could be used for risk detection (Ehrlich 2016). Current efforts are aimed at risk detection of chronic diseases, but given the association between gut dysbiosis and necrotizing enterocololitis, and the knowledge that certain bacterial strains appear more frequently in patients who develop NEC, this strategy could be applied to the disease in the future. At risk preterm infants would be good targets for microbiome analysis. Microbiome patterns thought to be associated with an increased risk for the development of necrotizing enterocolitis could then be ideal candidates for microbiome alteration.

## Conclusion

In summary, NEC is among the most common and lethal gastrointestinal diseases that plague premature infants and results in high short and long term morbidity and mortality. The pathogenesis of this complex disease has been studied for decades but only recently have advancements underscored the importance of the intestinal microbiome. The intestinal microbiome can have both a protective or a pathogenic role in preventing or contributing to the development of NEC. Achieving a healthy complement of commensal bacteria can help protect the preterm infant from gut inflammation and injury leading to necrotizing enterocolitis. Although much work lies ahead in order to translate the lessons learned in the laboratory to clinical practice, the manipulation of the intestinal microbiome is a promising strategy for the prevention of necrotizing enterocolitis.
